# Cochlear Implant Awareness: Development and Validation of a Patient Reported Outcome Measure

**DOI:** 10.3389/fnins.2022.830768

**Published:** 2022-04-29

**Authors:** Laura M. Markodimitraki, Inge Stegeman, Hans G. X. M. Thomeer

**Affiliations:** ^1^Department of Otorhinolaryngology and Head and Neck Surgery, University Medical Center Utrecht, Utrecht University, Utrecht, Netherlands; ^2^University Medical Center Utrecht Brain Center, University Medical Center Utrecht, Utrecht, Netherlands

**Keywords:** cochlear implants, cochlea, cochlear implantation, patient reported outcome measure (PROM), sensorineural hearing loss (SNHL), neurotology

## Abstract

**Background:**

Surgical success of cochlear implantation is usually measured through speech perception and quality of life questionnaires. Although these questionnaires cover a broad spectrum of domains, they do not evaluate the consciousness of wearing a cochlear implant (CI) and how this impacts the daily life of patients. To evaluate this concept we aimed to develop and validate a standardized patient reported outcome measure (PROM) for use in cochlear implant users.

**Methods:**

Development and evaluation of the COchlear iMPlant AwareneSS (COMPASS) questionnaire was realized following the COSMIN guidelines in three phases: (1) item generation, (2) qualitative pilot study to ensure relevance, comprehensiveness, comprehensibility, and face validity, and (3) quantitative survey study for the assessment of reliability (test-retest) with 54 participants.

**Results:**

Nine domains of CI awareness were identified through literature research and interviews with experts and patients. These resulted in the formulation of 18 items which were tested with a pilot study, after which 3 items were deleted. The final 15-item COMPASS questionnaire proved to have good validity and satisfactory reliability. The intraclass correlation coefficient calculated for items with continuous variables ranged from 0.66 to 0.89 with seven out of eight items scoring above the acceptable level of 0.7. The Cohen’s kappa calculated for items with nominal variables ranged from −0.4 to 0.78 with 11 (sub)items out of 15 scoring above fair to good agreement. Measurement error analysis for items with continuous variables showed a mean difference of −2.18 to 0.22. The calculated 95% limits of agreement for these items revealed no statistically significant difference between the two administered questionnaires. For items with nominal variables, the percentages of agreement calculated, ranged between 0 and 95%, and 83.3 and 96.6% for positive and negative agreement, respectively.

**Conclusion:**

The COMPASS questionnaire is a valid and reliable PROM for evaluating the cochlear implant awareness, and it can be easily used in routine clinical practice.

## Introduction

Cochlear implants (CI’s) are currently the only effective treatment for auditory rehabilitation for patients with severe-to-profound bilateral sensorineural hearing loss (SNHL) with poor speech perception. Since the introduction of this medical device in the 1970s, great advancements have been made regarding the functionality and hardware design. The internal part of the implant, the receiver/stimulator (R/S) device that resides under the skin behind the pinna of the ear, has undergone technological improvements resulting in thinner implants with smaller footprints (Carlson et al., 2012). Comfort of the external parts of the CI use has increased over the years with more discrete designs and lighter speech processors that allow patients to wear their implant throughout the day. Most importantly, the speech perception results have increased greatly, improving quality of life of patients with hearing loss (Gaylor et al., 2013; Loeffler et al., 2014; McRackan et al., 2018).

Despite the wealth of knowledge and research regarding speech perception results and health-related quality of life of CI recipients, little is known about the CI-experience and -awareness by patients. We define awareness of having a cochlear implant as “the state of mind or situation in which the patient is physically conscious he or she is wearing a cochlear implant and how this consciousness impacts their daily life.” There are patient-reported outcome measures (PROMs) assessing CI use such as the Cochlear Implant Management Skills (CIMS-self) survey and the Nijmegen Cochlear Implantation Questionnaire (NICQ) (Hinderink et al., 2000; Bennett et al., 2017). The CIMS-self focuses on device management exclusively, and the NICQ assesses health-related quality-of-life by how sound and speech perception limits a CI-recipient in their daily life. However, these PROMs do not evaluate the (physical) impact of a CI, thus they may fail to capture cochlear implant awareness topics in daily life that are of importance from patient perspective. To our knowledge, no CI-specific PROM has been developed yet that included patients in item development, following the standards of the Patient Reported Outcomes Measurement Information System (PROMIS) or the COnsensus-based Standards for the selection of health Measurement Instruments (COSMIN) (McRackan et al., 2018).

Cochlear implant awareness could be important for speech recognition results and quality of life of CI recipients. Studies have shown that wear time of the CI affects speech recognition outcomes in pediatric and adult patients (Gagnon et al., 2020; Holder et al., 2020). In addition, previous research on hearing aids has shown that fit and comfort are the second most important factors contributing to non-use of hearing aids (McCormack and Fortnum, 2013). Specifically, the satisfaction of patients with comfort of use, burden during daily activities, sleep disturbances related to location of the implant in relation to the preferred sleeping position, pain, or other discomfort caused by the implant are all contributing factors to reduced wear time. Moreover, there might be an underestimation of the prevalence of above mentioned problems in CI recipients, especially when a significant increase in hearing and communication is achieved using the CI. The benefits of the CI could suppress the concomitant inconvenience that accompanies wearing the processor.

In order to assess the physical awareness of the cochlear implant, we aimed to develop and validate a patient reported outcome measure (PROM) questionnaire.

## Materials and Methods

**FIGURE 1:**
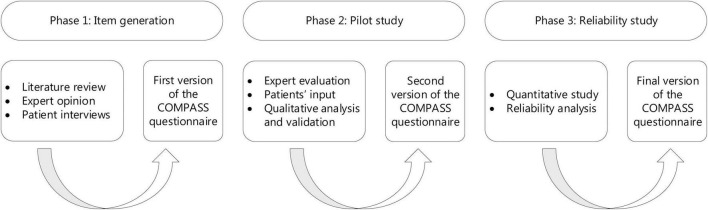
The three phase procedure for development and validation of the COMPASS questionnaire.

This development and validation study was conducted between December 2019 and April 2021 at the University Medical Center (UMC) Utrecht, in compliance with the principles of the Declaration of Helsinki. This study was exempt from approval of an ethics committee under Dutch law. Exemption was granted by the local ethical committee (Institutional Review Board of the UMC Utrecht) (METC protocol 19-722/C). A three-stage procedure for development and validation of the patient reported outcome measure (PROM) was conducted, in accordance with the COSMIN guidelines (see Figure 1; Mokkink et al., 2010). Participants were recruited at the time of routine control at the CI center UMC Utrecht, and through an open e-mail invitation to patients registered in the CI database of the UMC Utrecht sent by their attending physician. Written informed consent was obtained from all participants.

### Construction of the Concept

We aimed to develop the COchlear iMPlant AwareneSS (COMPASS) questionnaire to assess the awareness of having a cochlear implant as previously defined. The PROM development group consisted of a otorhinolaryngologist, an epidemiologist and a junior researcher. This questionnaire was designed for adult, Dutch speaking, CI recipients. The instrument was developed to be used as a self-administered evaluation tool, in daily clinical practice, for clinical studies, and for comparison within patients over time (possible evolution of awareness). The questionnaire was designed to detect issues in different categories, specifically concerning the external parts of the CI (speech processor and transmitter) and the internal part (the receiver/stimulator device). In order to assess CI awareness, different domains were identified. It is important to distinguish the situation of awareness and how burdensome the awareness is. Therefore, the questionnaire should consist of multiple choice items as well as scale items to measure the burden. With the results of the questionnaire, health care professionals should be able to identify problems that can be solved by adapting the hardware or by counseling.

### Phase 1: Item Generation

Qualitative data were obtained by a literature review, a series of interviews with seven specialists in cochlear implantation care, including an otorhinolarygologist, speech therapists and audiologists, and individual interviews with a sample (*n* = 7) of CI recipients were conducted, to identify and select relevant aspects of CI awareness. Included patients were adult CI recipients that were using their implant for at least 1 year prior to inclusion in order to have adequate experience with everyday use of their implant to contribute to data collection. The semi-structured interviews of approximately 1 h each were recorded and were conducted by a trained investigator (LM) using an interview guide (see Supplementary Material). The recorded interviews were then transcripted verbatim. Content analysis was performed independently by two researchers (LM and IS), by coding the transcripts and then grouping the codes into thematic categories. Data collection was continued until saturation was reached. The emerging domains as well as the pertinence of the findings were discussed within the research group until consensus was reached. The questionnaire is based on a formative model, the indicators (items) define the value of CI awareness (the construct measured).

### Phase 2: Pilot Study (Cognitive Debriefing Test)

A pilot study was conducted to assess the content validity of the questionnaire, the comprehensibility and comprehensiveness. The above mentioned experts in the field of cochlear implantation evaluated the content, wording, format, answer options, and intelligibility. Changes were made appropriately. The evaluated questionnaire was administered to ten adult CI patients that were using their implant for at least 3 months prior to inclusion in order to have adequate experience with everyday use of their implant to contribute to data validation. Participants filled out the questionnaire while “thinking aloud,” followed by a semi-structured interview with open-ended questions (see Supplementary Material) that were audio-recorded. This interview was conducted to capture information on the participant’s understanding of the instructions, the intended meaning and clinical relevance of each item, the response options, patients opinion regarding the questionnaire and missing concepts. The time required to fill out the questionnaire was also recorded. Adjustments were made to the questionnaire based on these interviews.

### Phase 3: Reliability Study

A quantitative study was conducted to assess the reliability of the final version of the COMPASS questionnaire. The questionnaire was administered twice to 54 adult CI patients, thereby meeting the COSMIN criteria of participants necessary for quantitative validation (Mokkink et al., 2010). These CI patients were using the CI for at least 3 months prior to inclusion in order to have adequate experience with everyday use of their implant to contribute to data validation. Two weeks after the participants filled out and returned the questionnaire, they were send and filled out the same questionnaire again. The questionnaire was distributed on paper or digitally through Castor EDC (version 1.6, Ciwit B.V., Amsterdam, Netherlands), an electronic data capture platform, depending on the patients’ preferences.

### Data Analysis

Data was analyzed using IBM SPSS Statistics for Windows (version 26.0.0.1; IBM Corp., Armonk, NY, United States). Reliability (test-retest) was calculated using the interclass correlation coefficient (ICC) for continuous scores and Cohen’s Kappa with standard error and 95% confidence interval for nominal scores. We used the two-way random effect model with interaction for the absolute agreement between single scores to calculate the ICC with 95% confidence interval. This model was chosen because time is a relevant factor for the test-retest assessment, and because the results will be generalized beyond the study points. Also the participants are assumed to be stable for the construct of interest across the two time points (Qin et al., 2019). Values > 0.70 are generally considered as good (Nunnally and Bernstein, 1994). However, the ICC should be interpreted with the sample variability in mind. Therefore, we calculate the range of scores per item to illustrate the homogeneity of the subjects. Small inter-subject variability results in a depress of the ICC (Weir, 2005). To interpret the values of kappa we used the criteria by Fleiss et al.: values < 0.40 represent poor agreement, 0.41–0.75 fair to good and ≥0.75 represent excellent agreement (Fleiss et al., 2003).

Measurement error, the systematic and random error of an individual patient’s score that is not attributed to true changes in the construct to be measured, was assessed by Bland-Altman plots with the 95% limits of agreement for continuous scores, and the positive and negative percentage agreement for nominal scores.

### Scoring the COMPASS

The final version of the COMPASS questionnaire consisted of 15 items. These were divided into two subdomains: external and internal device domains. The external device (speech processor and transmitter) domain and the internal device (receiver/stimulator) domain consisted of seven and eight items, respectively. Items were either multiple choice or visual analogue scale questions. Each item had a maximum score of 5, with a total maximum score of 75. A higher COMPASS score represented a higher awareness level.

## Results

### Phase 1: Item Generation

Domains of awareness that were identified through literature search were bulging of the implant under the skin, discomfort or pain caused by the implant and sleep disturbances related to the implant. Domains identified through expert interviews were pain caused by the speech processor and transmitter, problems with wearing glasses, satisfaction with the position of the transmitter on the head, and interference of the external implant with daily activities and with wearing head covers (such as helmets). These domains were all mentioned by patients during the interviews in addition to problems with the transmitter coil (magnet falling off or being too strong). These domains of awareness were included in the first draft of the questionnaire. The domains most frequently mentioned were pain caused by the speech processor and/or magnet (mentioned by five out of seven participants), fear or discomfort caused by the external implant falling off the ear, and feeling a bulge where the internal implant resides under the skin (both mentioned by four participants). In order to measure these domains, 18 items were formulated. These items assessed the presence of the domains contributing to awareness and the burden that it created for the patient. Eight dichotomous (yes/no) items assessed the presence of domains; one multiple choice item assessed the ideal position of the transmitter according to the patient; seven visual analogue scale (VAS) items assessed the burden of these domains and two VAS item assessed pain caused by the external parts of the CI and in the area of operation.

### Phase 2: Pilot Study (Cognitive Debriefing Test)

A pilot study was conducted with 10 CI patients (see Table 1 for characteristics of the participants). The mean time to complete the questionnaire was 5 min and 21 s (range 3:10–9:40). Based on the results of the item analysis and the cognitive debriefing test small revisions to the questionnaire items and response options were made to ensure comprehensibility and comprehensiveness. Four items measuring interference of the CI with daily activities that overlapped and two items measuring interference of the CI with wearing glasses were fused into two items, one multiple choice item including all activities that the CI could pose troubles with wearing glasses, and one visual analogue scale item measuring burden experienced by these problems. Two items assessing satisfaction with the position of the CI were removed that were deemed not specific for identifying the underlying issue that causes CI awareness. Thus the scoring results of these items would not be helpful for the clinician using this PROM. Two items assessing sleep disturbance caused by the implant were split into four items to increase specificity of the domain by assessing change of sleep position and awareness of the implant while lying on the operated side of the head. Lastly, one item was added to include more complaints other than pain, as suggested by the CI patients. Thus, the number of items was reduced to 15 (see Table 2 and Supplementary Figure 1). Additionally, the lay-out of the paper questionnaire was adapted based on the suggestions of the CI patients.

**TABLE 1:** Characteristics of study participants per study phase.

Characteristics	Phase 1	Phase 2	Phase 3
	*n* = 7	*n* = 10	*n* = 52
Age, mean (SD) [range]	68.6 (7.3) [62–80]	60.7 (14.3) [31–76]	65 (12.9) [18–82]
*Sex, No. (%)*			
Male	3 (42.9)	6 (60.0)	35 (67.3)
Female	4 (57.1)	4 (40.0)	17 (32.7)
*CI model, No. (%)*			
Cochlear	4 (57.1)	4 (40.0)	25 (48.1)
Advanced bionics	2 (28.6)	3 (30.0)	6 (11.5)
MED-EL	1 (14.3)	2 (20.0)	18 (34.6)
Oticon Medical	0	1 (10.0)	3 (5.8)
*Operation side*			
Right	5 (71.4)	3 (30.0)	26 (50.0)
Left	1 (14.3)	4 (40.0)	26 (50.0)
Bilateral	1 (14.3)	3 (30.0)	0
CI use (months), mean (SD) [range]	100 (88.0) [13–253]	56.9 (74.7) [3–220]	30 (44.1) [3–234]

**TABLE 2:** COMPASS questionnaire items, answer options, and scoring calculations (not original lay-out).

No	Items	Answer options	Scoring calculation
1.	When I wear headgear (hat/cap/helmet/head scarf), I have to remove the transmitter (magnet).	○ Yes ○ No (go to question 3) ○ Not applicable for me. I never wear head gear (go to question 3)	Yes: 5 points No/Not applicable: 0 points
2.	If yes, how bothersome do you find having to remove the transmitter?	Not bothersome Extremely bothersome 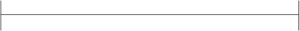	Visual analogue scale: 0–10 *Calculation:* score/2 = maximum 5 points
3.	The transmitter (magnet) sometimes falls off my head.	○ Yes ○ No (go to question 5)	Yes: 5 points No: 0 points
4.	If yes, how bothersome do you find that the transmitter (magnet) sometimes falls from your head?	Not bothersome Extremely bothersome 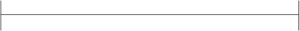	Visual analogue scale: 0–10 *Calculation:* score/2 = maximum 5 points
5.	The speech processor and transmitter (magnet) have inhibited me in the following activities: *(more than one option can be chosen)*	● Work ● Sport ● Transport (e.g., bicycling or driving) ● Social activities ● Wearing glasses (regular glasses/reading glasses/sunglasses) ● None of the above (go to question 7)	Each multiple choice item: 1 point None of the above: 0 points *Calculation:* Maximum 5 points
6.	If yes, how bothersome do you find that the speech processor and transmitter (magnet) inhibits you?	Not bothersome Extremely bothersome 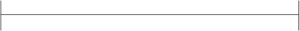	Visual analogue scale: 0–10 *Calculation:* score/2 = maximum 5 points
7.	When lying with my head on the operated side, I feel the cochlear implant under the skin.	Yes No (go to question 9)	Yes: 5 points No: 0 points
8.	How bothersome do you find that you feel the cochlear implant under the skin when lying on it?	Not bothersome Extremely bothersome 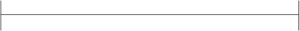	Visual analogue scale: 0–10 *Calculation:* score/2 = maximum 5 points
9.	I adjusted my sleeping position after the implantation because I want to avoid lying with my head on the operated side.	○ Yes ○ No (go to question 11)	Yes: 5 points No: 0 points
10.	If yes, how bothersome do you find adjusting your sleeping position.	Not bothersome Extremely bothersome 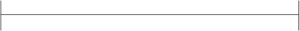	Visual analogue scale: 0–10 *Calculation:* score/2 = maximum 5 points
11.	I feel a protrusion where the cochlear implant resides under the skin.	○ Yes ○ No (go to question 13)	Yes: 5 points No: 0 points
12.	If yes, how bothersome do you find feeling a protrusion where the cochlear implant resides under the skin?	Not bothersome Extremely bothersome 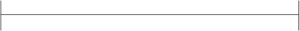	Visual analogue scale: 0–10 *Calculation:* score/2 = maximum 5 points
13.	How much pain have you had due to wearing the speech processor and the transmitter (magnet)?	No pain Unbearable pain	Visual analogue scale: 0–10 *Calculation:* score/2 = maximum 5 points
14.	I have had the following symptoms in the area of the operation. (*more than one option can be chosen)*	○ Pain ○ Numbness ○ Itchiness ○ None ○ Other: ___________________	Each multiple choice item: 1,25 points None: 0 points *Calculation:* Maximum 5 points
15.	*Fill out this question if you answered “Pain” in question 14. If not you can skip this question.* How much pain have you had in the area op operation.	No pain Unbearable pain 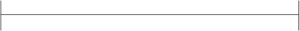	Visual analogue scale: 0–10 *Calculation:* score/2 = maximum 5 points
			Total score: maximum 75

*Disclaimer: This is a translation of the original Dutch questionnaire for the purpose of this manuscript only. Please refrain from using in the English language without validation. Instructions: With this questionnaire we aim to assess how much your life is affected in the last month by having a cochlear implant. Mark the answer that best resembles your situation, or click and hold the bar to move on the scale. Filling out the questionnaire will take approximately 10 min.*

### Phase 3: Reliability Study

We included 54 participants in the reliability study. A total of 52 participants (96.3%) filled out and returned both questionnaires. The unilaterally implanted study group had a wide age range (18–82 years) with an average age of 65 years (see Table 1 for demographics of the reliability study participants). Most of the population was male (67.3%). On average, the participants had been using the CI for 30 months (range 3–234 months).

Regarding the reliability analysis, the ICC, which represent reproducibility for the visual analogue scale items, ranged from 0.66 to 0.89 with only one item not meeting the acceptable level of 0.7, namely the item assessing the impact of the transmitter falling off the ear (see Table 3 for all ICC values with 95% confidence intervals). The Cohen’s kappa that was calculated for nominal items ranged from −0.4 to 0.78, with six (sub)items out of 15 scoring above fair to good agreement and five (sub)items scoring excellent agreement. The two multiple choice items (number five and fifteen), contained the four subitems that had poor agreement kappa values, with one subitem on inhibition of work due to the speech processor and transmitter scoring a negative value of −0.40 implying that there was no effective agreement between the two questionnaires on this item (see Table 4 for all Cohen’s kappa values with standard error and 95% confidence intervals).

**TABLE 3:** Reliability and measurement error analysis for visual analogue scale items (continuous data).

No	Items	Sample size	Reliability analysis		Mean difference (SD)	95% Limits of agreement	
			ICC	95% CI		Lower limit	Upper limit
2	Impact of taking off transmitter	17	0.73	0.24–0.9	0.15 (3.40)	−6.51	6.82
4	Impact of falling of transmitter	30	0.66	0.28–0.84	0.13 (3.50)	−6.73	6.99
6	Impact of speech processor and transmitter inhibiting activities	30	0.86	0.7–0.93	0.22 (2.08)	−3.85	4.29
8	Impact of feeling the cochlear implant under the skin	16	0.79	0.41–0.93	−0.72 (2.61)	−5.83	4.39
10	Impact of adjustment sleep position	5	0.84	−0.87–0.98	−2.18 (2.58)	−7.24	2.88
12	Impact of feeling the protrusion	41	0.88	0.78–0.94	−0.17 (1.34)	−2.79	2.46
13	Pain due to wearing the speech processor and the transmitter	52	0.89	0.81–0.94	−0.20 (0.97)	−2.10	1.70
15	Amount of pain in the operation area	8	0.83	0.1–0.97	0.26 (1.59)	−2.86	3.38

*Items are numbered in accordance with the COMPASS questionnaire.*

**TABLE 4:** Reliability and measurement error analysis for checkbox and multiple choice items (nominal data).

No	Items		Cohen’s kappa	Standard error	95% CI	Agreement (%)	
						Positive	Negative
1	Taking off transmitter to wear headgear		0.7	0.09	0.53–0.88	82.8	90.9
3	Transmitter falls of head		0.73	0.09	0.55–0.91	87.3	85.7
5	Speech processor and transmitter inhibiting activities:	i. Work	−0.40	0.02	−0.44–0.36	0	96.0
		ii. Sports	0.51	0.16	0.19–0.82	58.8	92.0
		iii. Transport	0.24	0.23	−0.22–0.69	28.6	94.8
		iv. Social activities	0.19	0.21	−0.22–0.60	25.0	93.8
		v. Glasses	0.63	0.12	0.39–0.86	73.3	89.2
		vi. None of the above	0.62	0.11	0.40–0.83	81.5	80.0
7	Feeling the cochlear implant under the skin while lying on it		0.76	0.10	0.57–0.96	82.8	93.3
9	Adjustment of sleep position		0.78	0.15	0.49–1.07	80.0	97.9
11	Feeling the protrusion of the cochlear implant		0.78	0.10	0.58–0.99	95.0	83.3
14	Symptoms in the area of operation	i. Pain	0.77	0.13	0.51–1.02	80.0	96.6
		ii. Numbness	0.77	0.13	0.51–1.02	62.5	96.6
		iii. Itchiness	0.56	0.16	0.24–0.87	87.9	93.2
		iv. None	0.67	0.11	0.46–0.88	61.5	94.5

*Items are numbered in accordance with the COMPASS questionnaire.*

The mean difference for items of continuous variables was −2.18 to 0.22. The 95% limits of agreement (LoA) revealed no statistically significant difference between the two administered questionnaires in all continuous variables (zero is included in each interval) (see Table 3). We observed higher mean differences with wider 95% LoA for items with smaller sample sizes (see Supplementary Figure 2 for Bland-Altman plots). Percentages of agreement ranges between 0 and 95%, and 83.3 and 96.6% for positive and negative agreement, respectively. The positive agreement percentage showed the widest range, with the multiple choice items number three and eight scoring the lowest values (see Table 4).

## Discussion

The purpose of the study was to develop and validate a PROM to assess CI awareness, thus the state of mind or situation in which the patient is physically conscious he or she is wearing a cochlear implant and how this consciousness impacts their daily life. The COMPASS questionnaire was developed following the COSMIN guidelines (Mokkink et al., 2010) for development of PROMs and was based on expert opinion and patient interviews, pilot tested with a cognitive interview study, and validated by administrating it to a population of CI recipients. We tested the content validity (comprehensibility, comprehensiveness, and relevance), and reliability of the questionnaire. The COMPASS questionnaire consists of 15 items and showed fair to excellent test-retest reliability for almost all items and measurement error analysis revealed no systematic or random errors of the score per patient. The lowest reliability and positive agreement scores were calculated for the activities impeded by the speech processor and transmitter; in particular work, transport, and social activities. This could suggest that any restrictions caused by the external part of the CI during these particular activities, varies over time, even in the short test-retest time period of 2 weeks.

We believe that prospective assessment of CI awareness using a PROM, can provide more accurate information on any existing problems. We know that hearing aid issues such as discomfort and handling problems, are common amongst users of these medical devices, one study reporting a prevalence of 98% (McCormack and Fortnum, 2013; Bennett et al., 2020). However, some patients might experience problems with their hearing aids, though do not report them to their clinician (Bennett et al., 2019). One study on cochlear implant recipient issues, reported that the majority of patients included in the study (89.8%), had at least one CI device handling problem (Bennett et al., 2017). Previous studies using patient reported outcome measures also found a high prevalence of other adverse events, such as change of taste. Lloyd et al. (2007) and Mikkelsen et al. (2017) reported changes of taste after surgery in 16.9 and 45% of CI patients, respectively. The COMPASS questionnaire could be used by clinicians to assess issues caused by the external and internal components of the CI that contribute to awareness of the cochlear implant. These issues could be solved by counseling or arranging accessories such as an adjustment of magnet power. Moreover, the location of the implant in relation to the ear pinna might be adjusted likewise (cap wearing interferes with superior implant positioning).

The questionnaire fills in the gap and responds to the needs of the implantees that experience negative effects of the presence of the subperiosteal implant. Cochlear implants have undergone tremendous developments in the last decades regarding shape, hardware volume and intrinsic technical refinements. The different manufacturers produce R/S device aspects that are quite divers. One of the interesting developments is the significant reduction in implant volume, that might decrease implant protrusion visible at the level of the skin. Moreover, this might prevent the surgeon to drill a bony well in the temporal cortex as beforehand with the older implant the gold standard has been to drill a well, to tackle this issue. To our knowledge, there is little evidence thus far available regarding the influence of implant volumes reduction or the effects of drilling or not drilling a bony well, on CI awareness of a patient and implant related complaints. Our developed questionnaire meets these goals. Items assessing burden by issues caused by the internal device such as protrusion of the skin, sleep disturbances due to the implant or problems with headgear, could be rectifiable post-implantation by revision surgery (and re-positioning the implant), however, it might be advisable to perform the implantation correctly during primary implantation. Therefore the COMPASS questionnaire could be used in clinic to assess the impact of different surgical methods for positioning and fixation of the R/S device on CI awareness.

A limitation of this study is the study population sample used for development and validation of the questionnaire, which was recruited from a single center. This could introduce selection bias, however, participants were operated by several CI surgeons with different surgical techniques. Also, assessment of the criterion validity of the COMPASS questionnaire could not be executed. After extensive literature research, we were unable to find validated outcome measures assessing CI use as defined in this study. Furthermore, despite our hypothesis that there are indeed differences of CI awareness between groups, it was impossible to execute this validation step. We expect that patients operated with different fixation techniques of the R/S device will differ in CI awareness. However, in our center we only use one fixation technique (the bony bed technique), and thus we could not compare these groups. Lastly, all four CI device brands were represented in the study population, and patients included in the study had sufficient experience with using the CI to contribute to the study.

In conclusion, the COMPASS questionnaire has good reliability and validity. Combining this PROM with clinical findings may assist in the routine follow up of patients with CI. Furthermore, it can be used as an endpoint in a clinical study, to evaluate different surgical techniques and its effect on awareness.

## Data Availability Statement

The raw data supporting the conclusions of this article will be made available by the authors, without undue reservation.

## Ethics Statement

The studies involving human participants were reviewed and approved by the local ethical committee (Institutional Review Board of the UMC Utrecht) (METC protocol 19-722/C). The patients/participants provided their written informed consent to participate in this study.

## Author Contributions

All authors contributed equally to draft and revised the manuscript and approved the final submission.

## Conflict of Interest

The authors declare that the research was conducted in the absence of any commercial or financial relationships that could be construed as a potential conflict of interest.

## Publisher’s Note

All claims expressed in this article are solely those of the authors and do not necessarily represent those of their affiliated organizations, or those of the publisher, the editors and the reviewers. Any product that may be evaluated in this article, or claim that may be made by its manufacturer, is not guaranteed or endorsed by the publisher.
