# Supplementary material for: Cochlear Implant Awareness: Development and Validation of a Patient Reported Outcome Measure
Source: Front Neurosci. 2022 Apr 29;16:830768. doi: 10.3389/fnins.2022.830768 (PMC9100413; doi:10.3389/fnins.2022.830768)
Supplement: Supplementary file 1 [file Data_Sheet_1.docx]

Supplementary Material

# Interview probe for Phase 1 interviews: item generation

- Have you had or do you currently have any issues with the use of the cochlear implant?
- Does wearing the cochlear implant cause you any (physical) problems?
- Are you aware of your cochlear implant while using it? If so, in which situations are you aware of it?
- Are there aspects of the hardware of the implant that you would change if that were possible?
- What information were you given during the preoperative consultation about how the implant looks like and how you operate it?
- Were there any aspects about the use of the implant that you would have wanted more information on?
- Are you satisfied with the position of the implant on your head?
- Does the internal part of the implant protrude from the skin? If so, does this bother you?

# Interview probe for Phase 2 interviews: Pilot study (Cognitive Debriefing Test)

- Could you describe what this questionnaire is about?
- Were the questions clear for you? If not, which questions were unclear and why?
- Did you understand the instructions for filling out the questionnaire?
- Do you feel there were any topics or aspects of wearing a cochlear implant that were missed by the questionnaire?
- What did you think of the way the questions are posed and the answer options?
- (*optional in case of a hesitation in filling out a specific question)* Could you explain what question (…) is about?
- Do you have any suggestions to improve the questionnaire?

# Supplementary Figures and Tables


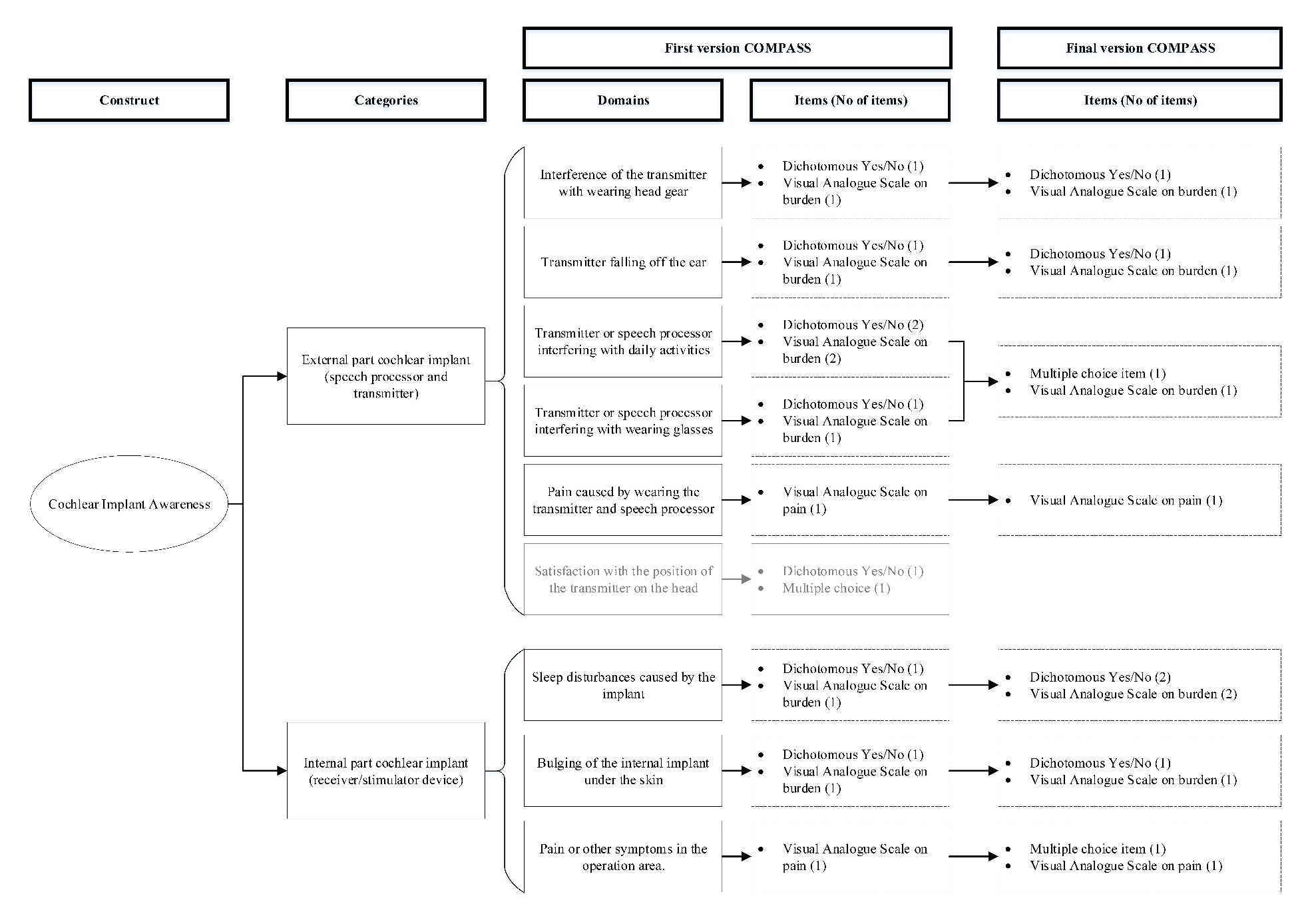
**Supplementary Figure 1.** COMPASS questionnaire domains and items per version

**Supplementary Figure 2.** Bland-Altman plot for the test-retest reliability. The plot illustrates the agreement between test and retest of the questionnaire and identifies possible outliers. Each sample is represented on the graph by conveying the mean value of the 2 assessments (x-axis) and the difference between the 2 assessments (y-axis). The mean difference was the estimated bias, and the standard deviation (SD) of the differences measured the fluctuations around this mean (outliers being above 1.96 SD diff ). Reference lines shows mean difference between time 1 and time 2 (solid line), and 95 % limits of agreement for the mean difference (broken lines)

| **ITEM 2** | 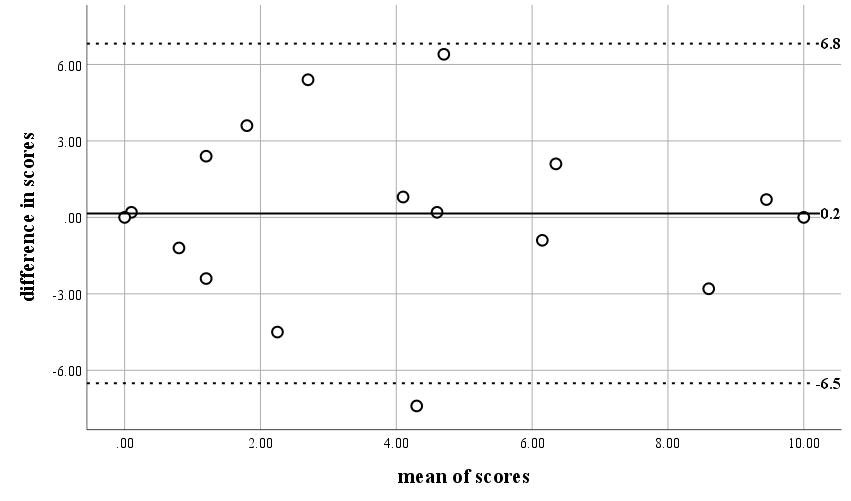 | **ITEM 4** | 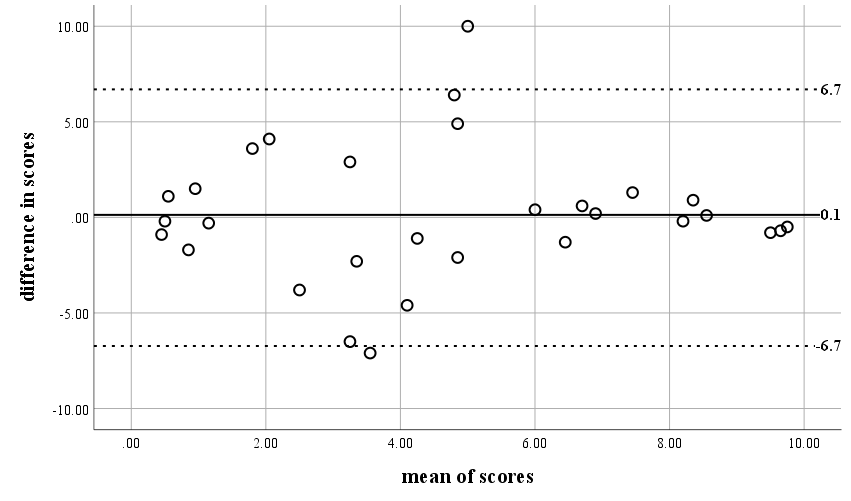 |
| --- | --- | --- | --- |
| **ITEM 6** | 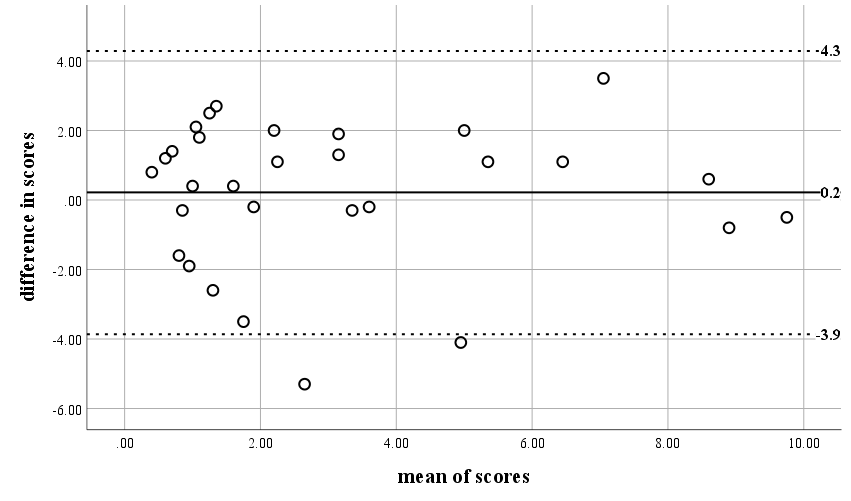 | **ITEM 8** | 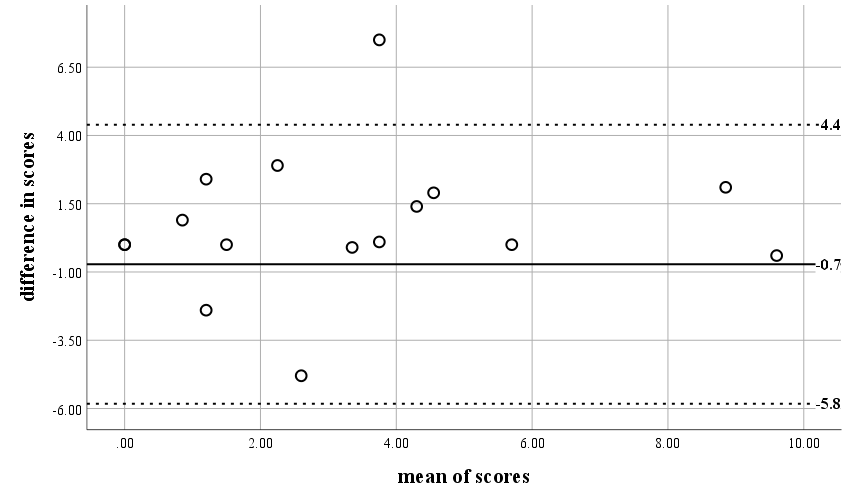 |
| **ITEM 10** | 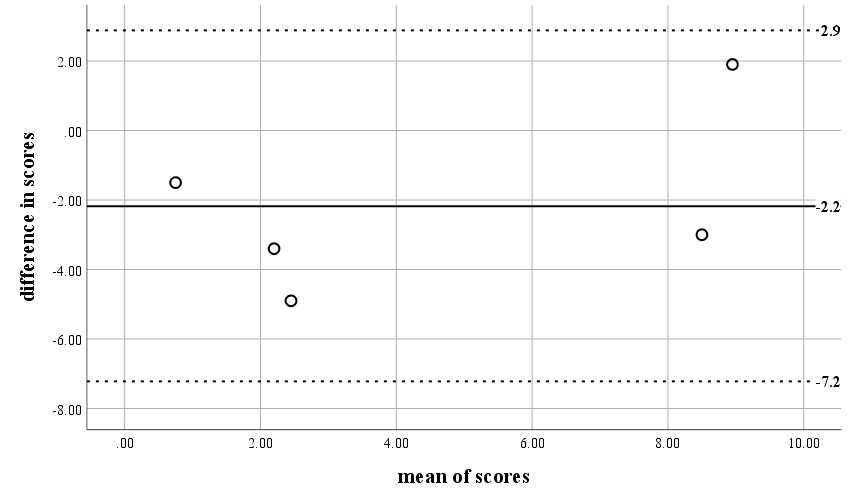 | **ITEM 12** | 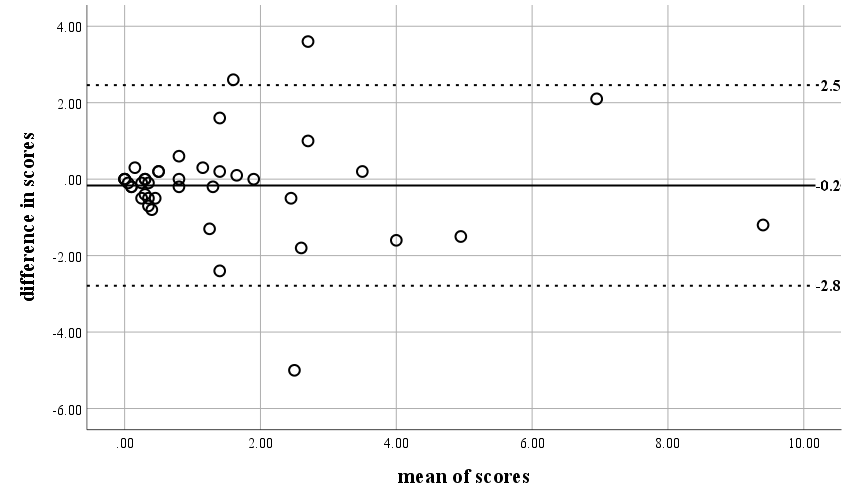 |
| **ITEM 13** | 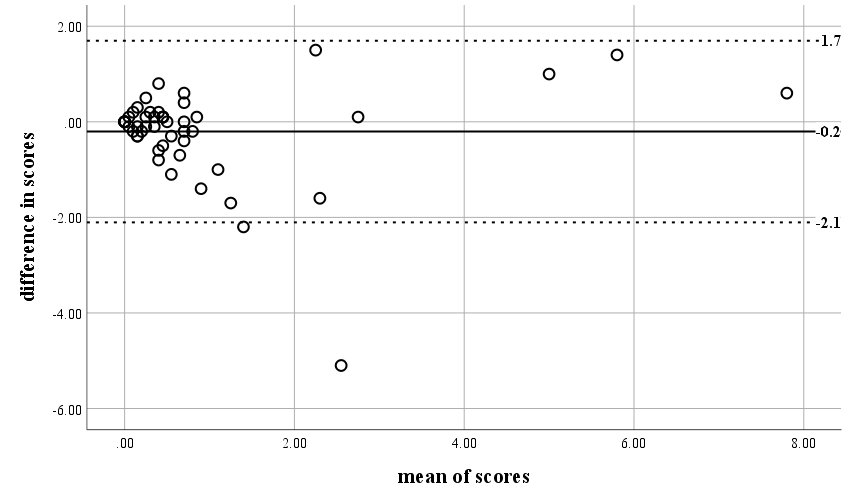 | **ITEM 15** | 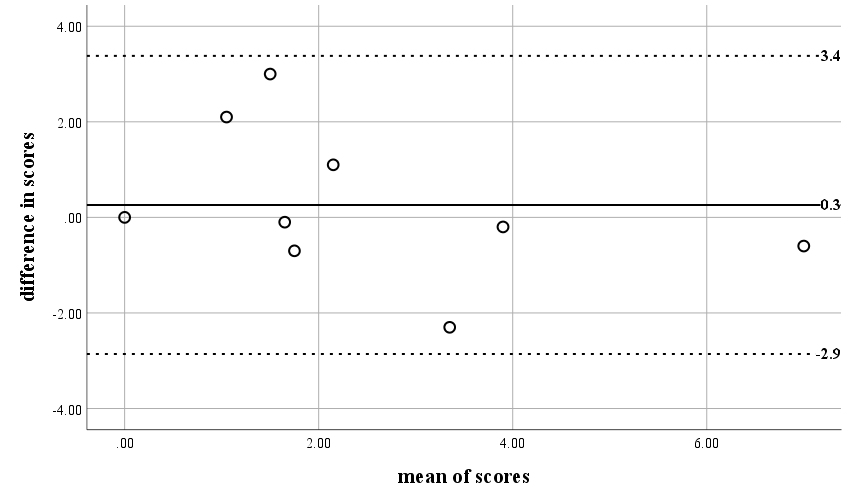 |
